# Severe Retinopathy of Prematurity Is Not Independently Associated With Worse Neurodevelopmental Outcomes in Preterm Neonates

**DOI:** 10.3389/fped.2021.679546

**Published:** 2021-06-10

**Authors:** Marie Altendahl, Myung Shin Sim, Artemiy Kokhanov, Bradley Gundlach, Irena Tsui, Alison Chu

**Affiliations:** ^1^Division of Neonatology and Developmental Biology, Department of Pediatrics, David Geffen School of Medicine, University of California, Los Angeles, Los Angeles, CA, United States; ^2^Department of Medicine, David Geffen School of Medicine, Statistics Core, University of California, Los Angeles, Los Angeles, CA, United States; ^3^Department of Ophthalmology, David Geffen School of Medicine, University of California, Los Angeles, Los Angeles, CA, United States

**Keywords:** Bayley-III, birth weight, neurodevelopmental outcomes, gestational age, retinopathy of prematurity, premature neonate, health insurance

## Abstract

**Purpose:** To evaluate the relationship between retinopathy of prematurity (ROP) severity and neurodevelopmental outcomes in premature neonates at 0–36 months corrected age.

**Methods:** A retrospective chart review was performed on 228 neonates screened for ROP at the UCLA Mattel Children's Hospital between 2011 and 2018. Demographic information, clinical outcomes, ROP severity (no ROP, type 1 ROP, type 2 ROP), and Bayley-III neurodevelopmental scores were collected. Infants were grouped into corrected age cohorts (0–12, 12–24, and 24–36 months) to assess neurodevelopmental outcomes with increasing age. Within each age cohort, ANOVA and Chi-Square testing were used to detect differences in birth characteristics and neurodevelopmental scores between infants with type 1 ROP, type 2 ROP, or no ROP. Univariable analyses assessed the relationship between ROP severity and neurodevelopmental outcomes within each age cohort. A multivariable analysis was then performed to determine if ROP severity remained significantly associated with worse neurodevelopmental scores after controlling for birth weight (BW), intraventricular hemorrhage grade (IVH), health insurance type, male sex, and age at Bayley testing.

**Results:** Without controlling for factors associated with prematurity, neonates with type 1 ROP had poorer cognition (*p* = 0.001) and motor (*p* = 0.006) scores at ages 0–12 months and poorer cognition (*p* = 0.01), language (*p* = 0.04) and motor (*p* = 0.04) scores at ages 12–24 months than infants without ROP, but no significant differences were detected at ages 24–36 months. After adjusting for BW, IVH, insurance type, male sex, and age at Bayley testing, ROP severity was no longer associated with worse neurodevelopmental scores in any domain.

**Conclusion:** This study emphasizes that poorer neurodevelopmental outcomes in preterm neonates are most likely related to lower birthweight, associated co-morbidities of prematurity, and socioeconomic factors such as health insurance, not severity of ROP itself.

## Introduction

Globally, ~11.1% of births are premature (1–3). Although advances in neonatal healthcare have improved outcomes for premature infants, they are still at risk of developing retinopathy of prematurity (ROP) and poorer visual outcomes later in life (4–6). Generally, the more premature or smaller a preterm baby is at birth, the higher their risk of developing more severe ROP. ROP is characterized by aberrant retinal blood vessel development (7, 8). In ROP, relatively elevated oxygen levels in preterm infants requiring oxygen therapy for immature lungs promote vascular attenuation and subsequently lead to retinal hypoxia (8, 9). This period of local hypoxia results in increased release of hypoxia-inducible factor 1 alpha (Hif1a) and vascular endothelial growth factor (VEGF), consequently stimulating pathological proliferation of retinal blood vessels, which in severe cases, can lead to traction on the retina, retinal detachment, and permanent blindness (8, 9).

ROP can be classified as type 1 or type 2, based on the Early Treatment for Retinopathy of Prematurity (ETROP) classification (10). Type 1 ROP is more severe than type 2 and requires treatment for ROP via laser photocoagulation and/or anti-VEGF therapy (10). Infants with type 2 ROP require close monitoring, and treatment may be considered if type 2 ROP is persistent past 52 weeks gestational age (10, 11). Infants with ROP, including those who have been treated, require long-term monitoring for the development of visual impairments, such as macular dragging, myopia, and strabismus (4, 12).

It has been proposed that ROP may lead to significant visual impairment which consequently portends worse later neurologic outcomes in infants and/or that pathological processes leading to ROP (such as intermittent hypoxia or oxidative stress) could also have detrimental effects elsewhere in the brain due to the similar embryological origins of both the eye and brain (13–15). To date, studies investigating the relationship between ROP severity and neurodevelopmental outcomes are conflicting (14, 16–21). Given the shared risk factors for neurological and visual impairment in preterm infants, the purpose of this study was to evaluate the relationship between ROP severity and neurodevelopmental outcomes at 0–12, 12–24, and 24–36 months of corrected age, while considering variables associated with prematurity known to portend worse neurodevelopmental outcomes [gestational age, birth weight, bronchopulmonary dysplasia (BPD), intraventricular hemorrhage grade (IVH), and socioeconomic status (SES)]. We hypothesize that worse ROP severity will not be associated with worse neurodevelopmental outcomes at any age after adjusting for the variables associated with prematurity.

## Materials and Methods

A retrospective cohort study was performed at the University of California, Los Angeles (UCLA) Mattel Children's Hospital on infants screened for ROP in the neonatal intensive care unit (NICU) between January 1, 2011 and December 31, 2018. The Institutional Review Board at UCLA approved the study protocol and granted waiver of consent.

### Study Participants

All neonates screened for ROP while hospitalized in the NICU at the UCLA Mattel Children's Hospital were eligible for the study. Infants eligible for ROP screening were identified by the neonatology team at UCLA. Study inclusion criteria were consistent with American Academy of Pediatrics (AAP) guidelines for ROP screening: infants born at a gestational age ≤30 weeks, birth weight <1,500 g, or gestational age at birth >30 weeks but with an unstable clinical course, such as infants on significant cardiorespiratory support (22). Participants who met AAP guidelines for ROP screening and completed at least one Bayley Scales of Infant and Toddler Development, third edition (Bayley-III) neurodevelopment assessment between 0 and 36 months of adjusted age were included in the study. Participants not meeting AAP guidelines for ROP screening or without available neurodevelopmental assessment data were excluded from the study.

### Demographic and Clinical Data

Demographic (sex, gestational age, and birth weight), clinical course/outcome information [fetal growth restriction (FGR), small for gestational age (SGA), BPD, and IVH], socioeconomic status information (insurance type), and visual outcomes (myopia, strabismus, amblyopia, and optic atrophy) were collected for each subject via electronic medical review. FGR was determined by the obstetric team through serial prenatal ultrasound. SGA was defined as a birth weight percentile <10% for gestational age and sex (23, 24). BPD was defined as the need for supplemental oxygen or respiratory support at 36 weeks gestational age. IVH grade was defined as the worst IVH grade on any postnatal ultrasound as interpreted by pediatric radiologists according to Papile grading (25). Socioeconomic data collected included health insurance type upon admission to the NICU. Health insurance was categorized as either public or private health insurance. Visual outcomes were determined by a pediatric ophthalmologist in outpatient follow-up. Myopia was defined as spherical equivalent refraction < -6 diopters in either eye. Amblyopia, strabismus, and optic atrophy diagnosed/documented by a pediatric ophthalmologist at any clinical visit were categorized as present or not present.

### ROP Screening

ROP screening was performed by board-certified pediatric ophthalmologists at the recommended intervals according to the 2013 AAP guidelines (22). Worst ROP stage, ROP zone, presence of plus disease, and need for interventional treatment (anti-VEGF or peripheral retinal ablation) were evaluated for each patient and data was collected via electronic medical review. For this study, participants were classified by their worst ROP stage as having no ROP, type 1 ROP (high-risk), or type 2 ROP (low-risk pre-threshold) as defined by ETROP classification (10). As such, infants were treated for ROP with peripheral retinal ablation or anti-VEGF for type 1 ROP. If infants had persistent Type 2 ROP beyond 52 weeks, peripheral laser was considered to minimize burden of follow-up.

### Outcomes—Neurodevelopmental Assessment

The primary outcome variables for this study were composite cognition, language, and motor domain scores assessed using the Bayley Scales of Infant and Toddler Development, third edition (Bayley-III) (26). Participants, at 0–36 months adjusted age, completed at least one formal neurodevelopmental evaluation (Bayley-III) assessed by a group of trained pediatricians, physical and occupational therapists, and clinical psychologists at the UCLA High Risk Infant Follow-up Clinic. Electronic medical record review was used to record participants' Bayley-III composite cognition, motor, and language scores and the participants' corrected age at examination. Due to the retrospective study design, infants' neurodevelopmental outcomes were evaluated at variable timepoints. For example, some infants had their first neurodevelopmental assessment before 12 months corrected age, but others were not assessed until later or records were not available until later ages. Given that Bayley-III assessments at older ages may be more predictive of school age outcomes (27) and to account for the variability in ages at which neurodevelopmental examinations were performed, we categorized neurodevelopmental exams into three corrected age groups: 0–12, 12–24, and 24–36 months. All statistical analyses were performed within each age group independently. If an infant had more than one evaluation completed within an age group period, the latest assessment score was used. If a participant did not have an evaluation completed during an age group, the participant was excluded from all analyses specific to that age group.

### Analyses

For each age group, differences in demographic, clinical and vision outcome data between ROP groups (no ROP, type 1 ROP, and type 2 ROP) were assessed using ANOVA (gestational age and birth weight) and Chi-Square Tests (sex, IVH, BPD, health insurance type, FGR, SGA, and vision outcomes). In univariable analysis, ANOVA was used to assess the association between ROP severity (no ROP, type 1, and type 2 ROP) and Bayley-III composite cognition, language, or motor scores within each age group. In multivariable analysis, the association was assessed between neurodevelopmental scores and infants with any ROP (type 1 or 2) vs. no ROP after controlling for variables found to be highly associated with neurodevelopmental scores. To account for the correlations among Bayley scores from each infant, we used a linear mixed effects model. The model selection steps involved backward eliminations and forward selections during which likelihood tests and Akaike information criteria were used for nested and un-nested model comparisons, respectively. Comparisons of neurodevelopmental scores of three different age cohorts (0–12, 12–24, and 24–36 months) were performed using the final mixed effect models and Tukey–Kramer's multiple comparison adjusted-*p*-values were used. To facilitate clinically meaningful interpretation of the analysis, we constructed a generalized linear mixed effect (Glimmix) model using the same factors identified from the mixed effect model. Odds ratios (OR), 95% confidence intervals (CI), *p*-values, and area under the receiver operating characteristics curves (AUROC) were generated from Glimmix models where the outcomes were whether or not patients had moderate to severely impairment by Bayley (cognitive, language, and gross Motor) scores defined by impairment more than two standard deviations (SD) below the mean, compared to no or mild impairment (Bayley score <2 SD below the mean). A 2-sided *p* < 0.05 was considered statistically significant and all analyses were performed using SAS 9.4 (Cary, NC).

## Results

Three hundred and sixty infants were screened for ROP exams at the UCLA Mattel Children's Hospital between January 1st, 2011 date and December 31st, 2018 date. One hundred and thirty-two infants did not have available data on neurodevelopmental assessments and were excluded from the study. The remaining 228 infants met study inclusion criteria and were included for analysis.

### Demographic, ROP, Vision Outcomes, and Neurodevelopmental Data

Our study cohort of 228 infants had a mean gestational age of 28.5 ± 2.8 weeks (range: 22.3–34.6 weeks) and mean birth weight of 1,089 ± 373 g (range: 470–2,370 g). One hundred and six (47%) infants were female, 94 (41%) infants had BPD, 70 (31%) infants had FGR, 38 (16.7%) infants were SGA, and 91 (40%) infants had IVH (grades I–IV). Eighty-eight (42.9%) neonates had public health insurance and 117 (57.1%) neonates had private health insurance. Twenty-three (10%) infants had type 1 ROP, 66 (29%) infants had type 2 ROP, and 139 (61%) infants had no ROP. Thirty-eight infants were treated for ROP; this included infants with type 1 ROP and those with persistent type 2 ROP (Table 1).

**Table 1:** Summary of demographic data and clinical outcomes for infants with no ROP, type 2 ROP, and type 1 ROP.

	**No ROP**		**Type 2 ROP**		**Type 1 ROP**			
	***N***	**Mean (*SD*)**	***N***	**Mean (*SD*)**	***N***	**Mean (*SD*)**	***F***	***p***
**Bayley age: 0–12 months**								
Gestational age (Weeks)	117	29.9 (2.00)	55	26.8 (2.51)	19	25.5 (2.08)	61.0	<0.0001^**^
Birth weight (g)	117	1249 (319)	55	872 (296)	19	758 (183)	42.0	<0.0001^**^
	***N***	***n*** **(%)**	***N***	***n*** **(%)**	***N***	***n*** **(%)**	***X***^**2**^	***p***
Sex (Female)	117	52 (44.4)	55	29 (52.7)	19	7 (36.8)	1.76	0.42
Diagnosis of BPD	117	30 (25.6)	55	36 (65.5)	19	14 (73.7)	33.1	<0.0001^**^
Diagnosis of IVH	117	36 (30.8)	55	27 (49.1)	19	11 (57.9)	8.55	0.014^*^
Diagnosis of FGR	117	37 (31.6)	55	17 (30.9)	19	6 (31.6)	0.009	0.99
SGA Status	117	21 (18.0)	55	9 (16.4)	19	3 (15.8)	0.099	0.95
Public health insurance	106	31 (29.2)	52	27 (51.9)	19	15 (78.9)	19.9	<0.0001^**^
**Bayley age: 12–24 months**								
Gestational age (Weeks)	86	29.8 (2.09)	40	25.9 (2.53)	17	25.6 (1.97)	56.1	<0.0001^**^
Birth weight (g)	86	1,277 (362)	40	780 (273)	17	745 (203)	42.0	<0.0001^**^
	***N***	***n*** **(%)**	***N***	***n*** **(%)**	***N***	***n*** **(%)**	***X***^**2**^	***p***
Sex (Female)	86	43 (50.0)	40	21 (52.5)	17	7 (41.2)	0.6	0.73
Diagnosis of BPD	86	21 (24.4)	40	32 (80.0)	17	11 (64.7)	37.2	<0.0001^**^
Diagnosis of IVH	86	23 (26.7)	40	24 (60.0)	17	9 (52.9)	14.2	0.001^**^
Diagnosis of FGR	86	23 (26.7)	40	12 (30.0)	17	5 (29.4)	0.2	0.92
SGA status	86	13 (15.1)	40	7 (17.5)	17	4 (23.5)	0.7	0.69
Public health insurance	71	25 (35.2)	36	19 (0.52)	17	13 (76.4)	10.3	0.006^**^
**Bayley age: 24–36 months**								
Gestational Age (Weeks)	28	29.7 (2.60)	22	25.9 (1.79)	9	24.6 (1.46)	27.3	<0.0001^**^
Birth Weight (g)	28	1291 (388)	22	771 (239)	9	827 (170)	19.1	<0.0001^**^
	***N***	***n*** **(%)**	***N***	***n*** **(%)**	***N***	***n*** **(%)**	***X***^**2**^	***p***
Sex (Female)	28	9 (32.1)	22	12 (54.6)	9	3 (33.3)	2.8	0.25
Diagnosis of BPD	28	5 (17.9)	22	16 (72.7)	9	5 (55.6)	15.6	<0.0001^**^
Diagnosis of IVH	28	6 (21.4)	22	10 (45.5)	9	8 (88.9)	13.2	0.001^**^
Diagnosis of FGR	28	7 (25.0)	22	6 (27.3)	9	0 (0.00)	3.0	0.22
SGA status	28	4 (14.3)	22	4 (18.2)	9	0 (0.00)	1.8	0.40
Public health insurance	24	12 (50.0)	18	14 (77.8)	9	6 (66.7)	3.5	0.18

*Describes results from ANOVA models assessing differences in risk factors between infants with no ROP, type 2 ROP, and type 1 ROP within each age group.*

* *p < 0.05,*

** *p < 0.01*.

One hundred and thirty-nine infants were seen for follow-up ophthalmology appointments. Out of these 139 children, 27 (19.4%) children had strabismus, 10 (7.2%) children had amblyopia, 8 (5.8%) children had optic nerve atrophy, 1 (0.7%) child had macular dragging, and 8 (5.7%) children had myopia. Rates of myopia, strabismus, and amblyopia were significantly different among infants with no ROP, type 1, and type 2 ROP (Table 2). However, there were no associations between rates of optic nerve atrophy and macular dragging amongst the ROP groups.

**Table 2:** Summary of visual impairment in 139 participants seen for eye examination at the University of California, Los Angeles.

	**No ROP**		**Type 2 ROP**		**Type 1 ROP**		
	***N***	***n* (%)**	***N***	***n* (%)**	***N***	***n* (%)**	***p***
Myopia	83	0 (0.0)	38	4 (10.8)	18	4 (22.2)	<0.0001^**^
Strabismus	83	10 (12.0)	38	10 (26.3)	18	7 (38.9)	0.015^*^
Amblyopia	83	0 (0.0)	38	5 (13.2)	18	5 (27.8)	<0.0001^**^
Optic nerve atrophy	83	2 (2.4)	38	3 (7.9)	18	3 (16.7)	0.050
Macular dragging	83	0 (0.0)	38	1 (2.63)	18	0 (0.0)	0.262
Any visual impairment	83	10 (12.0)	38	13 (34.2)	18	9 (50.0)	<0.0001^**^

*Compares the rates of myopia, strabismus, amblyopia, optic nerve atrophy, macular dragging, or any visual impairment between infants without ROP, type 1 ROP, and type 2 ROP (Chi-square). Infants with type 1 ROP had significantly higher rates of myopia, strabismus, amblyopia, optic nerve atrophy, and any visual impairment than infants without ROP or those with type 2 ROP.*

* *p < 0.05,*

** *p < 0.01*.

**Figure 1:**
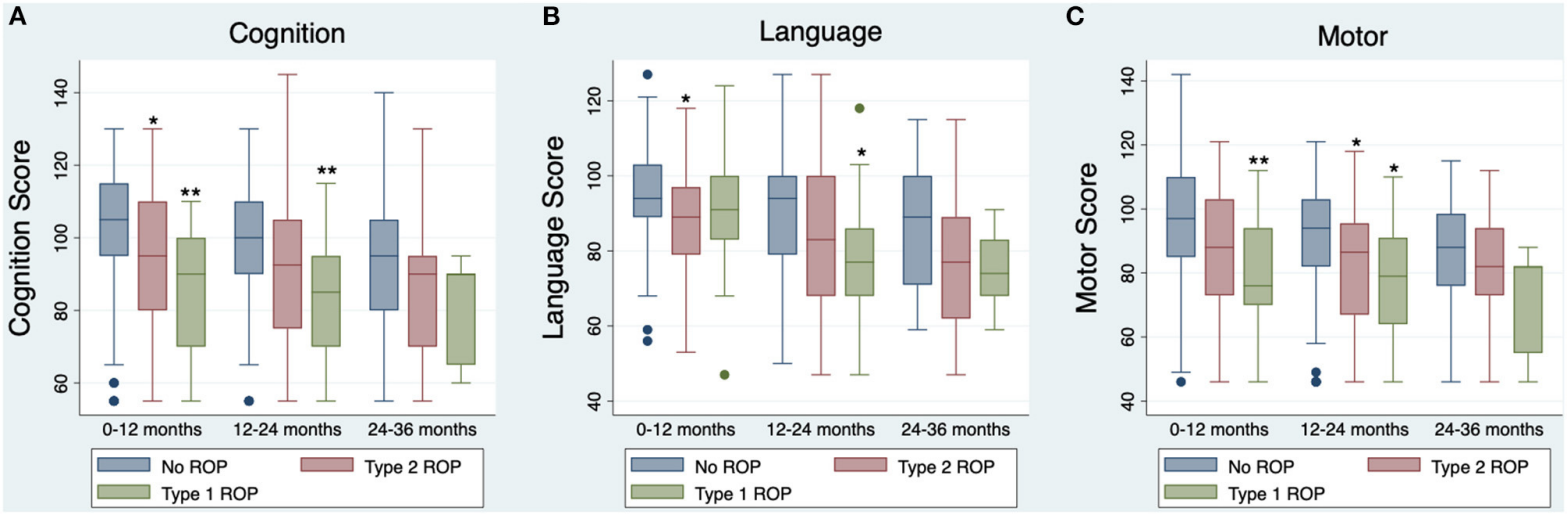
Boxplots of Bayley-III Neurodevelopmental Composite Scores, by univariable analysis (uncorrected for covariates). Boxplot lines and rectangles indicate the median and 1.5 × interquartile range below the first quartile or above the third quartile. The navy circles represent scores falling outside of that range, though all scores were included in analyses. Asterisks represent significant differences in Bayley-III neurodevelopmental scores between infants without ROP and infants with type 1 or type 2 ROP as measured by *post-hoc* analyses, *p < 0.05, **p < 0.01. **(A)** Cognition scores at 0–12 months (*n* = 191), 12–24 months (*n* = 142), and 24–36 months (*n* = 59) for infants with no ROP (blue), type 2 ROP (red), or type 1 ROP (green). **(B)** Language scores at 0–12 months (*n* = 191), 12–24 months (*n* = 142), and 24–36 months (*n* = 56) for infants with no ROP, type 2 ROP, or type 1 ROP. **(C)** Motor scores at 0–12 months (*n* = 191), 12–24 months (*n* = 142), and 24–36 months (*n* = 59) for infants with no ROP, type 2 ROP, or type 1 ROP.

Given that neurodevelopmental assessment data was available over 0–36 months, neurodevelopmental outcomes were grouped and assessed separately at three different time points. One hundred and niney-one infants completed neurodevelopmental assessments at 0–12 months corrected age, 142 infants completed neurodevelopmental assessments at 12–24 months corrected age, and 59 infants completed neurodevelopmental assessments at 24–36 months corrected age. Neurodevelopmental information for infants in each age group are represented in Table 3 and Figure 1.

**Table 3:** Summary of Bayley-III neurodevelopmental scores for infants assessed at 0–12, 12–24, and 24–36 months.

	**No ROP**		**Type 2 ROP**		**Type 1 ROP**			
	***N***	**Mean (*SD*)**	***N***	**Mean (*SD*)**	***N***	**Mean (*SD*)**	***F***	***p***
**0–12 Months**								
Cognition	117	101.0 (17.1)	55	93.8 (19.6)	19	85.0 (17.1)	8.05	0.0004^**^
Language	117	94.6 (12.9)	55	88.8 (13.3)	19	89.9 (16.7)	3.96	0.021^*^
Motor	117	95.2 (19.6)	55	87.9 (19.5)	19	80.2 (18.0)	6.29	0.002^**^
**12–24 Months**								
Cognition	85	97.7 (15.6)	40	91.3 (19.8)	17	84.1 (17.8)	5.30	0.006^**^
Language	85	90.1 (16.3)	40	84.2 (20.7)	17	78.4 (18.0)	3.78	0.025^*^
Motor	85	91.3 (18.1)	40	82.1 (19.0)	17	79.0 (20.7)	5.17	0.007^**^
**24–36 Months**								
Cognition	28	94.5 (18.9)	22	86.6 (18.0)	9	79.4 (15.3)	2.73	0.074
Language	26	88.0 (17.3)	21	77.6 (18.3)	9	75.6 (10.7)	3.00	0.058
Motor	28	84.7 (19.2)	22	80.1 (17.2)	9	72.0 (16.8)	1.71	0.191

*Describes the results from univariate analyses (ANOVA) comparing neurodevelopmental outcomes between infants with no ROP, type 1 ROP, and type 2 ROP.*

* *p < 0.05,*

** *p < 0.01*.

Fifty-nine children were seen for neurodevelopmental assessment at 24–36 months, which was less than the number of infants assessed at 0–12 and 12–24 months. Children identified as high-risk because of continued significant medical and developmental concerns which necessitate a higher level of care coordination continue to receive neurodevelopmental assessments after the age of 24 months at the UCLA High Risk Infant Follow-up Clinic, whereas children who are categorized as lower risk because of reassuring improvements in their medical conditions and neurodevelopmental testing scores are “graduated” from the clinic around 24 months of age. In our cohort, children seen for neurodevelopmental assessment at 24–36 months had higher rates of type 1 ROP and type 2 ROP compared to children not assessed at 24–36 months (*p* = 0.041). 15.3% of children screened for neurodevelopmental assessment at 24–36 months had type 1 ROP and 37.3% of children had type 2 ROP. Of the children not screened at 24–36 months, only 8.3% and 26.0% had type 1 and type 2 ROP, respectively.

Gestational age, birth weight, diagnosis of IVH, and diagnosis of BPD were significantly different between infants with type 1 ROP, type 2 ROP, or no ROP in all three age groups and insurance type was significantly different between infants with type 1 ROP, type 2 ROP, or no ROP at ages 0–24 months (0–12 months: gestational age *p* < 0.0001, birth weight *p* < 0.0001, IVH *p* = 0.014, BPD *p* < 0.0001, insurance *p* < 0.0001; 12–24 months: gestational age *p* < 0.0001, birth weight *p* < 0.0001, IVH *p* = 0.001, BPD *p* < 0.0001, insurance *p* = 0.006; 24-36 months: gestational age *p* < 0.0001, birth weight *p* < 0.0001, IVH *p* = 0.001, and BPD *p* < 0.0001; Table 1). Specifically, infants with more severe ROP (type 1) were born at earlier gestational ages, had lower birth weights, higher rates of IVH and more severe IVH grades, had a higher prevalence of BPD, and were more likely to have public health insurance. There were no group differences between sex, diagnosis of FGR, diagnosis of SGA in any age group, or insurance type at 24–36 months (0–12 months: Sex *p* = 0.42, FGR *p* = 0.99, SGA *p* = 0.95; 12–24 months: Sex *p* = 0.73, FGR *p* = 0.92, SGA *p* = 0.69; 24–36 months: Sex *p* = 0.25, FGR *p* = 0.22, SGA *p* = 0.40, and insurance *p* = 0.18; Table 1).

### Univariable Analyses

The relationships between ROP severity and Bayley-III composite cognition, language, and motor scores for each age group were assessed using ANOVA. ROP severity was related to worse Bayley-III cognition, language, and motor scores at ages 0–12 months (cognition: *p* = 0.0004; language: *p* = 0.021; motor: *p* = 0.002) and 12–24 months (cognition: *p* = 0.006; language: *p* = 0.025, motor: *p* = 0.007; Table 2). *Post-hoc* analyses showed that these significant models were driven by participants with type 1 ROP having worse cognition (*p* = 0.001) and motor scores (*p* = 0.006) than infants without ROP at 0–12 months, and worse cognition (*p* = 0.010), language (*p* = 0.043), and motor scores (*p* = 0.043) than infants without ROP at 12–24 months (Figure 1). Additionally, *post-hoc* analyses showed that infants with type 2 ROP had significantly worse cognition (*p* = 0.045) and language scores (*p* = 0.025) than infants without ROP at ages 0–12 months and had worse motor scores (*p* = 0.034) than infants without ROP at ages 12–24 months (Figure 1). Importantly, there were no significant associations between ROP severity and neurodevelopmental outcomes at 24–36 months (cognition: *p* = 0.074, language *p* = 0.058, motor: *p* = 0.191; Table 2).

When comparing Bayley-III composite scores between infants with any ROP (type 1 or 2) to infants without ROP, infants with ROP were significantly more likely to have lower cognition, language, and motor scores at 0–12 months (cognition: *p* = 0.001; language: *p* = 0.006; motor: *p* = 0.002) and 12–24 months (cognition: *p* = 0.004; language: *p* = 0.014; motor: *p* = 0.002). Neonates with any ROP were also more likely to have lower cognition (*p* = 0.039) and language scores (*p* = 0.017) at 24–36 months than those without ROP. Neonates with ROP did not have significantly different motor scores at 24–36 months (*p* = 0.15).

### Multivariable Analyses

In order to assess if ROP was independently related to neurodevelopmental outcomes after co-varying for risks associated with poor neurodevelopmental outcomes in premature infants, we performed multivariable analysis using linear mixed effects models (for continuous Bayley scores) and generalized linear mixed effect models (for dichotomized Bayley scores- moderate to severe impairment compared to no or mild impairment). Lower birth weight, higher IVH grade, male sex and public insurance were identified as independently associated with worse Bayley scores. Moreover, given that there were differences in Bayley scores based upon the age at testing, this variable was also included in the model (Supplementary Figure 1). In this model, having any ROP (type 1 or 2) was not significantly independently associated with worse neurodevelopmental outcomes (moderate or severe impairment) in the cognitive (*p* = 0.43), language (*p* = 0.44), or motor domains (*p* = 0.48).

**Figure 2:**
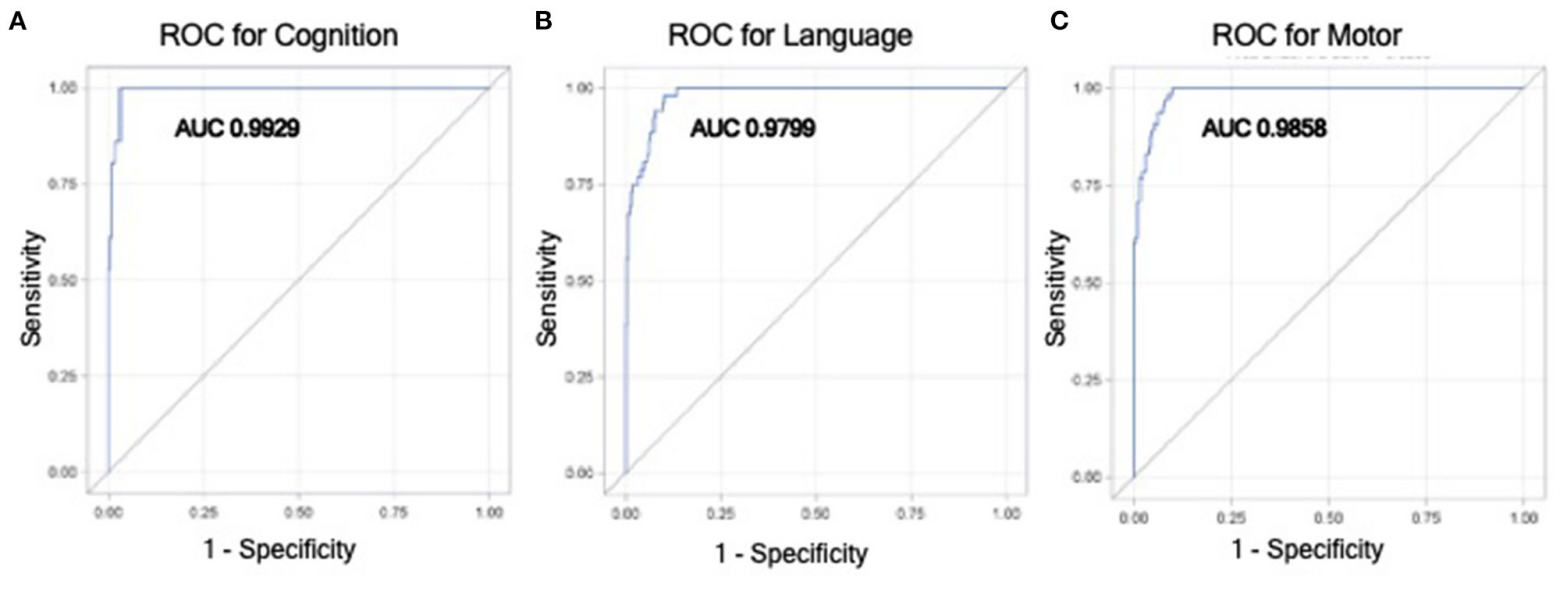
Receiver operator curves (ROC) from the generalized linear mixed effect model for having moderate to severe impairment vs. no to mild impairment in cognitive **(A)**, language **(B)**, and motor **(C)** domains. This model utilizes variables that were found to be significantly associated with worse neurodevelopmental outcomes in mixed effect modeling, which included birthweight, male sex, higher IVH grade, public insurance, and age at Bayley testing.

To provide clinically interpretable results, a Glimmix model for the dichotomized outcome of moderate to severe impairment vs. no or mild impairment was performed using the variables identified as significant in the multivariable mixed effect model (Table 4). For the cognitive domain, males infants or infants with IVH were ~14 × or ~8 × more likely to have moderate-severe impairment (male sex: *p* = 0.02; 0.0147). For the language domain, male infants were ~6 × more likely to have moderate-severe impairment (male sex: *p* = 0.007). As an infant's BW increased by 1 g, the likelihood of having a worse outcome was reduced by 0.002 (*p* = 0.020). For the motor domain, infants with private vs. public insurance were 8 × less likely to have worse neurodevelopmental outcomes (*p* = 0.007), infants with IVH were almost 5 × more likely to have worse outcomes (*p* = 0.021), and male infants were almost 9 × more likely to have worse outcomes (*p* = 0.005). These variables (male sex, BW, IVH grade, public insurance, and age at testing) were strong predictors for having worse neurodevelopmental outcomes, such that the AUCs for models including these variables for the cognitive, language, and motor domains were 0.993 (95% CI: 0.987, 0.999), 0.980 (95% CI: 0.968, 0.992), and 0.986 (95% CI: 0.977, 0.994), respectively (Figure 2).

**Table 4:** Summary of odds ratios and 95% confidence intervals for variables considered in the generalized linear mixed effect (Glimmix) model for each Bayley domain.

	**Cognition**	**Language**	**Motor**
	**OR (95% CI)**	**OR (95% CI)**	**OR (95% CI)**
Insurance (*Private vs. Public*)	0.320 (0.050–2.042)	0.246 (0.067–0.898)^*^	0.124 (0.027–0.558)^**^
Age at Assessment (*with each increment of 0–12, 12–24, and 24–36 months*)	1.172 (0.557–2.466)	2.795 (1.501–5.205)^**^	1.070 (0.604–1.894)
BW (*with each 1 g increase*)	0.998 (0.995–1.001)	0.998 (0.996–1.000)^*^	0.998 (0.996–1.000)^*^
IVH (*yes vs. no*)	7.961 (1.147–55.244)^*^	1.927 (0.593–6.263)	4.755 (1.266–17.859)^*^
Sex (*male vs. female*)	14.2 (1.788-113.247)^*^	6.358 (1.692-23.888)^**^	8.663 (1.921-39.073)^**^

*OR, odds ratio; CI, confidence interval.*

* *p < 0.05,*

** *p < 0.01*.

## Discussion

In summary, this study found that having ROP is not associated with worse neurodevelopmental outcomes as assessed by Bayley developmental testing after adjusting for important factors associated with prematurity-related poor neurodevelopmental outcomes (birthweight, IVH, male sex, and insurance status). These results confirmed our hypothesis that poorer neurodevelopmental outcomes in preterm neonates are most likely related to co-morbidities related to younger gestational age at birth and socioeconomic determinants of premature birth, not severity of ROP itself.

Counseling parents and caregivers of preterm neonates on the long-lasting effects of ROP can be challenging (28). Though ROP has been strongly associated with poorer vision, it previously was unclear how ROP might impact neurodevelopmental outcomes (14, 16–21). Allred et al. (29) reported that after controlling for gestational age and birth weight, participants with severe ROP in infancy were more likely to score two to three standard deviations below the mean on neurodevelopmental assessments (Bayley-III) than their less-severe ROP counterparts at 24 months corrected gestational age. Similarly, Glass et al. (15) found that participants with severe ROP in infancy had significantly lower cognition and motor scores (Bayley-III) at 18 months, and cognition scores remained significantly lower after controlling for gestational age and white matter injury (15). In contrast, Beligere et al. (13) detected no significant associations between severity of ROP by stage and neurodevelopmental impairment assessed using the OR Project skills inventory in children clinically followed by the Aravind Eye Care System. Stephenson et al. (21) similarly found that ROP severity did not relate to gross motor or cognitive performance in children 11–14 years of age (21). It is apparent from the variability in previous studies' results that the impact of ROP on neurodevelopmental outcomes later in childhood lacks a clear consensus. In our cohort, we report outcomes from a generally more recent cohort than in previous studies, and our finding that ROP is not associated with worse neurodevelopmental outcomes may also reflect improvements in neonatal care in the last decade.

Interestingly, studies showing significant relationships between ROP and worse neurodevelopmental outcomes often evaluated neurodevelopmental performance earlier in childhood than those that did not demonstrate an association (15, 20, 21, 29). For example, Glass et al. (15) and Allred et al. (29) found that infants with severe ROP had lower neurodevelopmental performances on Bayley Scales of Infant Development II or III at 18 and 24 months, respectively. Additionally, Drost et al. (20) found that at 15 months, participants with ROP in infancy had lower developmental quotients assessed by the Griffiths Mental Development Scale than matched controls, but there were no longer significant differences in cognition or gross motor assessed by Bayley-III at 24 months. Stephenson et al. (21) further emphasized that ROP did not predict cognitive or neurodevelopmental performance in late childhood. In their study of 198 children aged 11–14 years, participants with severe ROP did not perform worse on cognitive testing than those without ROP. Our data support these findings that developmental testing at younger ages (generally, before 2 years of age) are less reliable in predicting long-term neurodevelopmental disability; in our univariate analyses assessing how ROP severity predicted neurodevelopmental outcomes without co-varying for perinatal risk factors, we found that at ages 24–36 months, more severe ROP no longer predicted worse cognitive, language, nor motor performance. Our multivariate analysis, which accounted for age at developmental testing, also did not demonstrate an association of ROP severity with worse Bayley scores. We hypothesize three potential reasons for these results in our study. First, the number of participants in the 24–36 month age group decreased to 59 subjects, potentially resulting in insufficient power to detect differences. Second, infants still receiving neurodevelopmental assessment after 24 months in our high-risk follow-up clinic are those infants with the most significant medical and developmental concerns. This potential selection bias may enrich this group for infants without ROP who received neurodevelopmental assessment at 24–36 months because of underlying co-morbidities such cerebral palsy, which may have resulted in their poorer neurodevelopmental performance. This would be supported by the lower neurodevelopmental performances demonstrated across all infants aged 24–36 months in our cohort, regardless of ROP severity, potentially explaining why we no longer find a significant relationship between ROP severity and neurodevelopmental outcomes at 24–36 months. Lastly, as previous research shows, our results may represent that ROP does not accurately predict neurodevelopmental outcomes at later ages. Clinically, these findings emphasize the importance of referring ex-premature neonates for development follow up and interventional therapies, such as physical and occupational therapy, as early as possible after discharge to optimize attainment of developmental milestones in the first 2 years.

In our study population, ROP severity did not relate to neurodevelopmental outcomes at 0–36 months corrected age after co-varying for birth weight, IVH, male sex, and insurance type. A previous study by Glass et al. had a similar study design and found that infants with severe ROP had poorer Bayley-III cognition and motor scores at 18 months corrected age, after controlling for gestational age. Differences in categorizing ROP severity may explain the conflicting findings. Glass et al. (15) grouped infants into two categories (severe ROP or non-severe ROP) based on who required laser treatment for ROP. In contrast, our study grouped infants into three categories: type 1 ROP, type 2 ROP, or no ROP, based on ETROP guidelines. Although the majority of infants receiving laser treatment have type 1 ROP, some infants with persistent type 2 ROP may also be treated for ROP with laser surgery. Therefore, categorizing by stage of ROP disease rather than by need for ROP treatment may explain the difference in results. Of note, our multivariate analysis, which evaluated infants with any ROP vs. those with no ROP did not demonstrate worse neurodevelopmental scores. Additional studies by Schmidt et al. (18, 19) utilized univariate analyses to show that ROP is related to worse neurodevelopmental performance at both 18 months and 5 years corrected age. These results are potentially confounded by factors associated with prematurity, such as early gestational ages, low birth weights, IVH, BPD, and socioeconomic determinants of health outcomes (18, 19). Our results support the findings by Drost et al. (20) and Stephenson et al. (21): more severe ROP does not relate to poorer neurodevelopmental outcomes at 0–36 months corrected age, after controlling for the co-morbidities related to extreme prematurity.

The limitations of this study include that this is a single center retrospective study, and the small sample size and potential selection bias of the infants assessed at 24–36 months (*n* = 59). As stated above, children only receive neurodevelopmental assessments after the age of 24 months at the UCLA High Risk Infant Follow-up Clinic if they have continued significant medical and developmental concerns necessitating a higher level of care coordination. This resulted in the sample size of participants with neurodevelopmental testing between 24 and 36 months to be much lower than those at 0–12 months (*n* = 191) and 12–24 months (*n* = 142). Further studies on larger cohorts of ex-premature infants at school age would be helpful in addressing this limitation of our current study.

Currently, one of the most challenging aspects for clinicians working in the NICU is counseling parents on the likelihood of neurodevelopmental impairment in preterm neonates diagnosed with ROP. Our results emphasize that ROP is not associated with worse neurodevelopment performance at 0–36 months corrected age after adjusting for co-variates known to be associated with worse neurodevelopmental outcomes in preterm infants and despite infants with ROP having more visual impairments. Our study supports the overarching theme that the more premature and lower birth weight a neonate is/has, the higher risk they are for medical co-morbidities, including ROP, as well as worse neurodevelopmental outcomes. However, the co-morbidity of ROP itself does not appear to contribute to neurodevelopmental impairment.

## Data Availability Statement

The raw data supporting the conclusions of this article will be made available by the authors, without undue reservation.

## Ethics Statement

The studies involving human participants were reviewed and approved by the Institutional Review Board at UCLA. The UCLA IRB granted waiver of consent.

## Author Contributions

IT, AC, and MS created study design and statistical methods. AK and MA collected data. MA, BG, and MS performed statistical analyses. MA wrote the manuscript. MA, IT, AC, and MS contributed to data interpretation and critical revision of the manuscript. All authors contributed to the article and approved the submitted version.

## Conflict of Interest

The authors declare that the research was conducted in the absence of any commercial or financial relationships that could be construed as a potential conflict of interest.

## Supplementary Material

Supplementary Figure 1 Average Bayley scores in infants assessed at 0–12, 12–24, and 24–36 months. Bars represent least square means with standard errors estimated from the mixed effect model adjusting for insurance, sex, BW, and IVH.
