# Supplementary figures and images for: Severe Retinopathy of Prematurity Is Not Independently Associated With Worse Neurodevelopmental Outcomes in Preterm Neonates
Source: Front Pediatr. 2021 Jun 10;9:679546. doi: 10.3389/fped.2021.679546 (PMC8224761; doi:10.3389/fped.2021.679546)

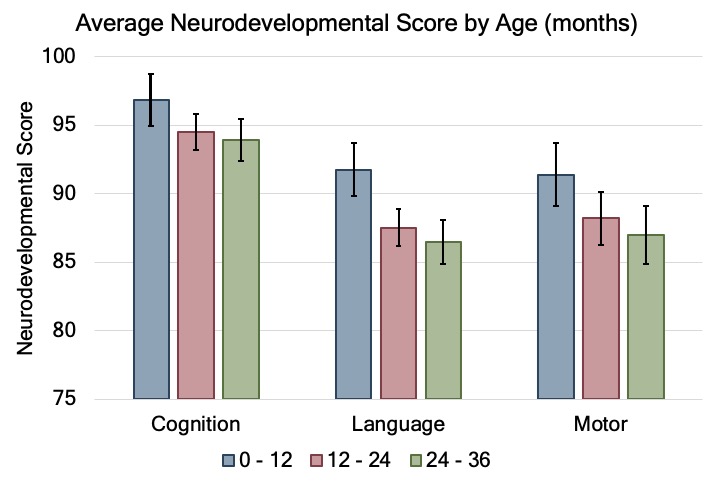

Supplement: Supplementary Figure 1 — Average Bayley scores in infants assessed at 0–12, 12–24, and 24–36 months. Bars represent least square means with standard errors estimated from the mixed effect model adjusting for insurance, sex, BW, and IVH. [file Image_1.JPEG]
